# Anterior cerebral artery aneurysm rupture presenting as hemorrhage in the splenium of the corpus callosum

**DOI:** 10.1590/0100-3984.2015.0130

**Published:** 2016

**Authors:** Thiago Giansante Abud, Andrew D. Nguyen, Lucas Giansante Abud, Emmanuel Houdart

**Affiliations:** 1 Department of Interventional Neuroradiology, Hospital Israelita Albert Einstein, São Paulo, SP, Brazil.; 2 Division of Neuro-Interventional Radiology, University of California-San Diego, San Diego, CA, USA.; 3 Department of Neuroradiology, Documenta - Hospital São Francisco, Ribeirão Preto, SP, Brazil.; 4 Department of Interventional Neuroradiology, Hôpital Lariboisière, Paris, France.

*Dear Editor*,

A 43-year-old, right-handed male presented with a three-day history of severe, holocranial headache. Three weeks prior, he had experienced another series of severe, pulsatile headaches accompanied by fever, malaise, and paresthesia of the second and third digits of the left hand. The neurologic examination revealed apraxia of the left hand and constructional apraxia of the right hand, without sensorimotor or cerebellar deficits, consistent with callosal disconnection syndrome.

**Figure 1:**
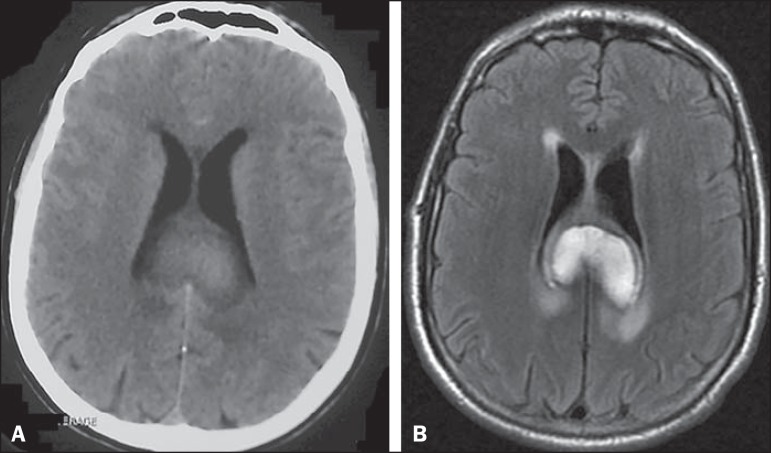
Non-contrast computed tomography **(A)** and T2-weighted fluid attenuated inversion recovery magnetic resonance imaging **(B)** demonstrating a large, heterogeneously enhancing mass in the splenium of the corpus callosum, consistent with a focal collection of intraparenchymal blood. No evidence of subarachnoid hemorrhage is apparent.

**Figure 2:**
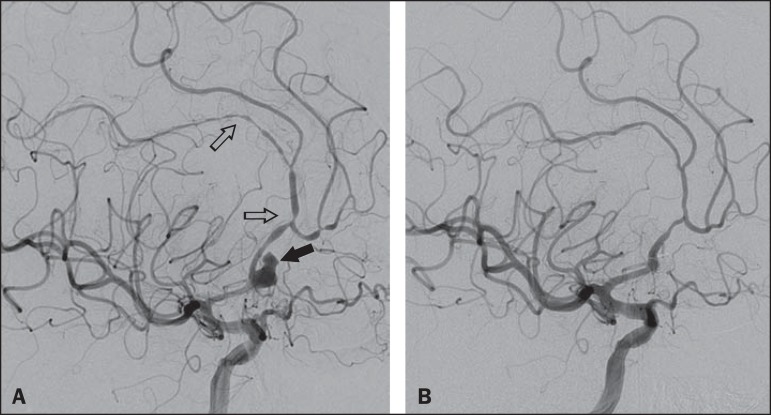
**A:** Digital subtraction angiography of the cerebral vessels, demonstrating an aneurysm (black arrow) at the junction of the A1 and A2 segments of the right ACA. The aneurysm is irregular in appearance, with a rupture sac and Murphy's test suggestive of recent rupture. The right A2 segment is characterized by an irregular caliber and a beaded appearance (open arrows), consistent with arterial vasospasm. **B:** Complete embolization of the aneurysm sac after coiling.

Non-contrast computed tomography and magnetic resonance imaging demonstrated a hematoma in the splenium of the corpus callosum (Figure 1). No subarachnoid blood was visualized. Cerebral angiography revealed evidence of recent aneurysm rupture at the junction of the A1 and A2 segments of the right anterior cerebral artery (ACA) and vasospasm of the distal right ACA (Figure 2A). The decision was made to embolize the aneurysm with detachable coils (Figure 2B). At the conclusion of the procedure, there was complete embolization of the aneurysm sac, without disruption of the integrity of the intracranial arteries or defect in the brain parenchyma. The remainder of the hospital stay was uneventful, and the patient was discharged on post-admission day 11 with a prescription for a 6-day tapered course of nimodipine. Angiography performed at 6 months of follow-up demonstrated that the coils remained in place within the aneurysm sac (i.e., the aneurysm sac continued to be occluded).

Reports of remote intraparenchymal hemorrhage as a presenting finding of aneurysm rupture are rare^(1)^. For example, in a group of 460 patients with subarachnoid hemorrhage, Abbed et al.^(2)^ reported 116 cases of intraparenchymal hematoma formation, none of which appeared to be proximal to the site of aneurysm rupture. In fact, our search of the literature revealed only isolated cases of remote focal hemorrhage. In 2002, Friedman et al.^(3)^ described a ruptured anterior communicating artery aneurysm associated with a perisylvian frontotemporal hematoma. Also in 2002, Lee et al.^(4)^ described the case of a patient with ruptured saccular ACA aneurysm that evolved to hemorrhage of the left putamen. In 2005, Paus et al.^(5)^ reported an even more perplexing case of anterior communicating artery aneurysm rupture, with adjacent subarachnoid hemorrhage and focal hematoma in the left posterior temporal lobe that was distant from the aneurysm and from any subarachnoid cisterns.

The case presented here is important because it establishes a mechanism for remote bleeding. In previous reports, a variety of explanations for distant hemorrhage have been proposed, including hypertensive crisis, the formation of jets through subarachnoid cisterns, venous infarction, intraluminal thrombosis, hemorrhagic infarction secondary to vasospasm, and occult vascular anomaly. However, none of those reports provided direct evidence to support any of the proposed mechanisms. In contrast, in our case, we observed definite angiographic evidence of vasospasm in the vessels between the aneurysm and the site of hemorrhage. That constitutes a strong indication that vasospasm-associated hemorrhagic infarction is a mechanism of remote hematoma formation following cerebral aneurysm rupture.

In conclusion, we have described a case of ACA aneurysm rupture presenting as remote intraparenchymal hemorrhage in the splenium of the corpus callosum and have demonstrated that vasospasm-induced hemorrhagic infarction is a plausible mechanism for distant bleeding. Neuroradiologists and neurosurgeons should be aware of this rare phenomenon in order to reduce the likelihood of inappropriate treatment.
